# The Efficacy of the Tabby Improved Prevention and Intervention Program in Reducing Cyberbullying and Cybervictimization among Students

**DOI:** 10.3390/ijerph15112536

**Published:** 2018-11-13

**Authors:** Anna Sorrentino, Anna Costanza Baldry, David P. Farrington

**Affiliations:** 1 Department of Psychology, Università degli Studi della Campania “Luigi Vanvitelli”, 81100 Caserta, Italy; anna.sorrentino1@unicampania.it; 2 Institute of Criminology, University of Cambridge, Cambridge CB3 9DA, UK; dpf1@cam.ac.uk

**Keywords:** cyberbullying, cybervictimization, prevention program, tabby intervention program, risk factors, threat assessment, ecological system theory

## Abstract

*Background*. This article presents results from the evaluation of the Tabby Improved Prevention and Intervention Program (TIPIP) for cyberbullying and cybervictimization. TIPIP is theoretically designed to address cyberbullying and cybervictimization. It is the first program in this field developed combining the Ecological System Theory and the Threat Assessment Approach. *Method*. The Tabby Improved program was evaluated using an experimental design with 759 Italian students (aged 10–17 years) randomly allocated via their classes to either the Experimental or Control Group. *Results*. Repeated measures ANOVAs showed a significant decrease both in cyberbullying and cybervictimization among students who received the intervention with a follow-up period of six months. The program was more effective for boys than for girls. *Conclusions*. Because cyberbullying is a cruel problem negatively affecting those involved, validated interventions that prove their efficacy in reducing the problem using experimental designs should be widely tested and promoted, paying particular attention to implementing a program fully to increase and guarantee its effectiveness.

## 1. Introduction

In the first decade of the 21st century, parallel to the development and the dissemination of the new communication technologies especially among youngsters, a new phase in school bullying studies started [1]. Researchers began to show interest in harmful behavior involving the use of information and communication technologies (ICTs) and the possible consequences, resulting in what has been since then identified as cyberbullying [2,3].

Cyberbullying is an increasing problem, involving students of all ages from many countries [4,5,6,7]. Several studies have found that involvement in cyberbullying is associated with negative behavioral, psychological, and psychosomatic outcomes for both cyberbullies and cybervictims [5,6,8,9]. Because of such negative consequences, studies have multiplied, and several cyberbullying prevention programs have been developed, implemented and tested [4].

Compared to the four decades of research on school bullying, research on the effectiveness of cyberbullying prevention programs is relatively recent. This means that effective and successful school bullying prevention strategies are known [5,10,11,12,13], but, to date, only a limited number of studies have evaluated the effectiveness of cyberbullying and cybervictimization prevention programs [14].

Based on the meta-analyses carried out by Mishna et al. [14], Tokunaga [15] and Slonje, Smith and Frisén [16], it is clear that although several cyberbullying prevention programs have been developed and evaluated, few have been specifically conceptualized for the prevention of cyberbullying by adopting sound theoretical frameworks. As pointed out by Tokunaga [15] and Slonje, Smith and Frisén [16], most programs are not based on any theoretical framework and there is a doubt whether they are effective, or if they are, why, and which component is working. Tanrikulu [6], in his systematic review of seventeen studies, assessing the effectiveness of cyberbullying prevention and intervention programs, found that four of these programs were not theoretically based while the other thirteen had very little or no theoretical background, making it difficult to understand the underlying theoretical structure of such programs and the criteria for using one or another component.

Regarding studies that assess the effectiveness of cyberbullying and cybervictimization prevention programs, consistent with Palladino et al. [17], we can identify three main types of programs designed to: (1) prevent school bullying that have been subsequently adapted to prevent cyberbullying; (2) specifically deal with cyberbullying; and (3) prevent both school bullying and cyberbullying. The KiVa program (Kiusaamista Vastaan) and the ViSC Social Competence Program, originated respectively in Finland and Austria for preventing school bullying, were then adopted and extended to address cyberbullying and cybervictimization. The KiVa program proved to be effective in reducing both cyberbullying and cybervictimization [18,19], while the ViSC Social Competence Program [20] was effective in reducing cyberbullying but not cybervictimization.

Regarding other programs, the *MedienHelden* (Media Heroes), developed in Germany by Schultze-Krumbholz, Wölfer, Jäkel, Zagorscak and Scheithauer [21], includes as its components empathy training and peer-to-peer tutoring on Internet safety, as well as teacher and parent training. This program proved to be effective in decreasing cyberbullying incidents [22,23]. The Spanish “*ConRed*” cyberbullying prevention program [24,25] also showed a significant reduction for both cyberbullying and cybervictimization. The Australian “*Cyber Friendly School Program*” [26] was assessed longitudinally and the results indicated a significant decrease in students’ likelihood of being involved in both cyberbullying and cybervictimization between the pre-test to the post-stages However, no significant differences were found between the intervention and control groups with regard to cyberbullying or cybervictimization. The “*Noncadiamointrappola!*” (*Let’s not fall into the trap*!) Program [27] consists of a peer-led approach to prevent both school bullying and cyberbullying. Evaluations of its effectiveness have shown a significant decrease in both cyberbullying and cybervictimization [17,27,28].

Many of these programs are limited as they only include a few components (e.g., a curriculum and training for teachers and/or activities with students) [29], and evidence about how many and which components are most relevant in preventing and reducing cyberbullying and cybervictimization is still scarce [5].

The present study aimed to overcome some of the above-mentioned limitations, by presenting results of the effectiveness on the so-called Tabby Improved Prevention and Intervention Program (TIPIP). This program is the first prevention and intervention program developed specifically for cyberbullying and cybervictimization by combining two sound theoretical frameworks: The Ecological System Theory [30,31] and the threat assessment approach [32,33,34,35].

### 1.1. Theoretical Background of the Program

Adopting and combining Bronfenbrenner’s Ecological System Theory (EST) and the Threat Assessment Approach seems to be a promising way to understand, explain and prevent youth involvement in cyberbullying and cybervictimization, deriving its roots from other fields of antisocial behavior and social psychology [36,37]. The EST [30,31] provides a comprehensive theoretical framework of the extent to which an individual’s involvement in cyberbullying and/or cybervictimization is affected by several factors: the students’ involvement, their families, peers, school, and community. The threat assessment approach [32,33,34,35] helps us to recognize and evaluate the presence of those risk factors that the international literature suggests are significant for students’ involvement in cyberbullying and cybervictimization [36].

The Threat Assessment Approach (TAA) [32,33,34,35] is applied to understand how to best prevent a threat for antisocial behaviors to occur. In the field of cyberbullying and cybervictimization, based on certain risk factors and needs, how likely is that these conducts will take place or will take place again in the future? The TAA was developed to address the threats taking place before a massive disaster (e.g., a shooting) or other violent act (e.g., in the workplace) could take place, to intervene in time.

Cyberbullying is characterized by many threatening behaviors and attitudes that might or might not result in attacking a child/adolescent online. The Ecological Systems Theory [30,31], on the other hand, allows for identifying the levels (individual, interpersonal, social, community) where those risk factors are, and by influencing each other, they could increase the risk of involvement of a certain individual in these aggressive behaviors. To this aim, dimensions identified by reviewing the international literature as risk factors for cyberbullying and/or cybervictimization were classified accordingly to the ecological systems identified by Bronfenbrenner [30,31].

By adopting this classification, it is possible to look at the relationship between risk factors and involvement in cyberbullying and cybervictimization [32,33,34,35] and evaluating the presence of risk factors at one or more of the four ecological levels identified by Bronfenbrenner [30,31,36] and assess the individual likelihood of being at risk.

The Tabby Improved Prevention and Intervention Program (TIPIP) has been designed to assess not only the presence of risk factors for cyberbullying and cybervictimization, but to identify the ecological levels in which those risk factors operate and interact with each other, in the directing of assessing future risk of any threatening circumstances (risk factors) taking place.

### 1.2. The Tabby (Threat Assessment of Bullying Behavior among Youngsters) Improved Prevention and Intervention Program (TIPIP)

**Figure 1:**
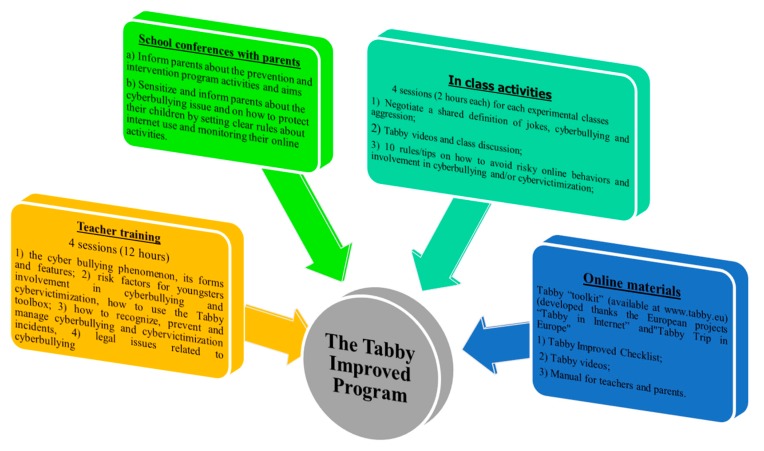
Components of the Tabby Improved Prevention and Intervention Program (TIPIP).

The TIPIP program was developed in two previous projects [4] and then ‘improved’ after evaluating the two previous projects’ results and validating the instruments and by analyzing the results derived by the review of the international literature on risk factors for youngsters’ involvement in cyberbullying and cybervictimization [36]. The program has four main components: (i) training activities with teachers, (ii) school conferences with parents; (iii) online materials for students, teachers and parents (available at www.tabby.eu); and (iv) in-class activities with students (see Figure 1).

Each of them is included in the program according to the Ecological System Theory [30,31] to address relevant dimensions involved in cyberbullying and cybervictimization prevention, that is the individual, his/her family, the peer group and the school by raising and increasing their awareness about risk factors for the involvement in those behaviors [32,33,34,35].

(i) The teacher training activities lasted three days, approximately three hours per session, once a week for three weeks, plus an additional day on the possible civil, criminal and administrative legal implications of cyberbullying and on age of responsibility. The training was scheduled as follows: (a) the cyberbullying phenomenon, its forms and features, similarity to and differences from school bullying; (b) risk factors for youngsters’ involvement in cyberbullying and cybervictimization, how to use the Tabby toolbox (the checklist, the booklet and the videos); (c) how to recognize, prevent and manage cyberbullying and cybervictimization incidents; (d) legal issues related to cyberbullying.

(ii) The School conferences with parents were scheduled in each of the participating schools. The main aims of these conferences were to: (a) inform parents about the prevention and intervention program activities and aims, and (b) sensitize and inform parents about the cyberbullying problem and how to protect their children by setting clear rules about internet use and how to best monitor their online activities. (iii) The third component of the program is the Tabby “toolkit” [4,38]. This is an extensive combination of three tools, including: the first consists of the updated version of the online self-report questionnaire, the Tabby Improved checklist, used to measure risk factors for students’ involvement in cyberbullying and cybervictimization; the second includes) four short videos, used as stimuli to make youngsters think about the cyberbullying phenomenon and its consequences. Each video addresses one of the most common types of cyberbullying and aims to increase youngsters’ awareness about the risks they face when using the Internet and the new communication technologies in a distorted or inattentive way. The central theme in each of the four videos is the idea that there is always an alternative, which helps to avoid either getting into trouble or causing trouble. For this reason, at the end of each video, after each cyber scenario, the story ‘rewinds’, showing what would or could have happened if the character(s) in the video had opted for another alternative (desirable) possible choice. At the end of the rewind scene, some recommendations on the safe use of the web are provided. Finally, the third tool is a manual for teachers, parents and students with useful information on cyberbullying, consisting of several short chapters with definitions and some scientific information on cyberbullying, is also a guide for trained teachers for them to organize class groups’ activities to raise students’ awareness about cyberbullying and cybervictimization. All are available at www.tabby.eu.

In-class activities with students were organized in each of the participating schools by scheduling four sessions (2 h each) for each of the experimental classes. The sessions with students were scheduled as follows: first, a group work aimed at negotiating a shared definition of jokes, cyberbullying and aggression. Once each group had defined these phenomena, they then had to identify differences and similarities between them. At the end of this activity, a representative from each group read to the class what emerged from their group discussion. Then all students chose the best definitions. The most highly voted work was exhibited in the classroom so that all students could share the same definitions of jokes, aggression, and cyberbullying. Then, during the second meeting, the Tabby videos described above were used. The videos were used as a stimuli from which to start a guided discussion regarding students’ experiences in the cyberspace and to discuss useful strategies to protect themselves and/or to put an end to cyberbullying and/or cybervictimization incidents. Then in a third meeting, students were again divided into small working groups. Each group had to prepare at least ten rules or tips on how to avoid risky online behaviors and involvement in cyberbullying and/or cybervictimization. Students were also asked to think about *rules* that they would comply to, and that the whole class would then be able to adopt as new rules. These *rules* drawn up by the experimental classes were then presented to the school principal. At the end of the project, these rules were disseminated to the whole school, and they were included in the participating schools’ policies on cyberbullying. In the fourth meeting, students had the opportunity to learn more about the legal consequences related to cyberbullying. During this last meeting a young boy who had been cyberbullying in the past met all classes to share his story and explain his point of view, answer questions and discuss what made him realize the damage caused by his actions and what he was doing to address it to change

The present study aimed to validate the effectiveness of the TIPIP by comparing pre and post and Experimental vs. Control Groups involvement in cyberbullying and cybervictimization.

## 2. Method

### 2.1. Design and Procedure

Five schools located in the Campania region, South Italy, participated in the project. Students were randomly assigned to one of two conditions (Experimental vs. Control), via their classes. Classes had to be randomly allocated to the research conditions because none of the contacted schools agreed to participate as a pure control school. To avoid possible teacher selection bias or class bias, the first author did the random assignment to the study conditions. Possible contamination effects were controlled by the first author, who was present and coordinated all the activities scheduled with students of the Experimental Group. In particular, most of the activities carried out with students were group work where students interacted with each other to understand what cyberbullying is, the dynamic and its impact and how to distinguish a credible and risky threat from a ‘joke’ or something that is not potentially harmful.

Before the data collection, the authors had obtained research approval from the Departmental Ethics committee for the procedure (29/2015), and consent from the custodial adults, and the participating students. All students filled in the Tabby online questionnaire before and six months after the whole intervention stages (T1 and T2). The first data collection was scheduled within the third month from the beginning of the school year, and then the procedure varied according to the condition (Experimental Group = EG, Control Group = CG); only students, teachers and parents of the EG received the intervention. The second data collection (follow-up T2), took place after six months, a few weeks before the end of the same school year.

To collect data, regardless of the research condition, the first author approached students in their classes, and, class by class, they all went with the teachers to the Computer Technology Room (CTR) that all participating school had to fill in the online questionnaire. Here, each student sat in front of a PC connected to the website questionnaire page and received instructions on how to proceed. Students were told that they should fill in an online, anonymous self-report questionnaire regarding their experiences on the use of new communication technologies and their online experiences, referring to a time period of six months.

Before filling in the questionnaire, the first author briefly explained the meaning of term cyberbullying so to make sure they had a common understanding of the main topic of the questionnaire and understood how their answers would not be available to anyone but collected and analyzed in an aggregated way. The following definition was provided:

*“Cyberbullying as an aggressive and intentional act, carried out by a group or an individual, using electronic forms of contact, repeatedly over time against a victim who cannot easily defend himself/herself”*.

[39]

Students were then instructed on how to generate an individualized ID code following a procedure to allow them to anonymously match their answers provided on that day (T1) with those on the following data collection (T2). The rule provided students was as follows: “Insert your personal code (two numbers of your date of birth- for example 03if you were born on the 3rd the, last two letters of your surname, and the last 3 numbers of your mobile or home phone number/if you don’t have it, e.g., 03BA362, for Barba born on the 3rd, with mobile nr: ++362). After completing the questionnaire, all students returned to their classes.

Only classes in the EG participated in the next stages of the study, which included the teacher training on cyberbullying (trained teachers did not teach in any of the classes assigned to the control condition); the in-school conferences with parents, and the class activities with students. Six months after the end of all stages of the intervention, students were again brought in the CTR fill in the same online questionnaire, this time referring to what had happened in the last six months. Students, at the beginning of the T2 questionnaire had to fill inn their ID following the same rule. Only matched students ended up in the final sample. Data for the Control Group were collected during the same period as for the EG.

### 2.2. Participants

The initial sample consisted of 759 students randomly recruited from five schools (49 classes). Classes were randomly divided into two groups corresponding to the conditions: 20 classes (40.8%) were in the Experimental Group (students who received the intervention), and 29 classes (59.2%) were in the Control Group (students who did not receive any intervention, but filled in the Tabby Improved Checklist).

Overall, 622 students were included in the analyses as they had taken part and completed phases T1 and T2 (82% of the initial sample) and their questionnaire could be correctly matched. Attrition rates were checked, and analyses did not show any significant differences in any of the variables investigated. The dropping out of just over one hundred students was namely due to mistakes in filling in the matching ID code that students themselves had to create to guarantee their anonymity or absence on the day of data collection. Of all students, 45.9% were males, (54.1% females), with an age range between 10 to 17 years old (M = 12.14, SD = 1.44).

With regard to the use of cyber communication, 29.4% of all students reported at least one profile on a social network. Of those who had a profile, 7.2% personally knew only half of their online contacts and 35.7% of students on average spent 2–4 h a day online (see Table 1 for details). With regard to students’ experiences of cyberbullying and cybervictimization, 15.0% reported cyberbullying others least once in the past six months, and 43.9% being cybervictimized at least once in the past six months. Boys were more likely to be cyberbullies but not cybervictims (see Table 1).

More boys were also reported having parents who did not talk with them about Internet security more than girls, control more their online activities, r spending more time per day on the Internet, being involved in both school bullying and victimization, and having poorer academic achievement.

### 2.3. Measures

The online Tabby Improved checklist was developed by analyzing the results of a review of the international literature on risk factors for youngsters’ involvement in cyberbullying and cybervictimization and how these risk factors operate and interact at different levels according to the ecological theoretical framework, and the short-term predictive ability of the risk the previous instrument [4,38].

The Tabby Improved checklist consists of 12 scales and a total of 130 items; for the purposes of the current study only certain dimension were used. All dimensions were selected to measure ontogenetic, microsystem, and community level risk factors. For the purpose of the present paper, two different scales were analyzed: Involvement in cyberbullying and cybervictimization (in the past six months). For both cyberbullying and cybervictimization, participants’ previous involvement in these behaviors was measured adopting the taxonomy by Willard [40]: flaming (A ‘*flame*’ is a deliberately hostile and provocative message sent from one user to the community or an individual. Flaming is done by sending violent or vulgar electronic messages, in order to arouse verbal conflicts within the network between two or more users), denigration, impersonation, outing, and exclusion (5 items for cyberbullying and 5 items for cybervictimization for each scale). Students rated their experiences of cyberbullying and cybervictimization on 5-point Likert scales ranging from 0 = “it has never happened in this period” to 4 = “it happened several times a week”. Example items: ‘I pretended to be someone else, created a fake profile in order to send or post damaging messages about another person’, ‘I disclosed online private information or images without the person consent’, and ‘I was actively engaged in excluding someone from an online group’.

At the end of the cyberbullying and cybervictimization items, students were asked about their involvement as cyberbullies and cybervictims in the past six months, using a final global question only used as a check item (‘In the last six months, have you ever been involved in cyberbullying?’).

To measure cyberbullying and cybervictimization, scores on the 5-items measuring different types of cyberbullying and cybervictimization were added, total scores ranged from 0 to 20. Reliability coefficients at T1 and T2 were respectively α = 0.64 and α = 0.75 for cyberbullying and α = 0.72 and α = 0.71 for cybervictimization. Even if the reliability coefficient of the cyberbullying measure at T1 was just sufficient values <0.60 are considered acceptable given the short scale dimension [41,42,43].

### 2.4. Analysis

Data analyses were carried out using the SPSS statistical package (version 21.0, IBM Milano, Milan, Italy). First, the possible differences between the Experimental and the Control Groups with regard to cyberbullying and cybervictimization pre-intervention measures were analyzed. Next, the Intraclass Correlation coefficients for the outcome measures (ICC) [44] were calculated. Because of the clustered randomization design of the study, the presence of any clustering effects could lead to an inaccurate test for statistical significance [45]. This analysis takes into account the possible similarity of the responses of individuals within each cluster (classes). Finally, to evaluate the impact of the program, we used repeated-measures ANOVAs. We analyzed the longitudinal differences (pre- and post-intervention) in cyberbullying and cybervictimization between the Control and Experimental Groups.

## 3. Results

The main scores revealed non-significant differences between the Experimental and the Control Group with regard to cyberbullying (F_(6)_ = 0.56, *p* > 0.05) and cybervictimization (F_(15)_ = 1.67, *p* > 0.05) measured at baseline (T1). Because of the clustered randomization design of the study, the Intraclass Correlation coefficients for the dependent measures were calculated, obtaining ρ = −0.002 for the cyberbullying pre-test score and ρ= −0.001 for the cybervictimization pre-test score. These results indicated that clustering effects were very small and negligible compared with the 0.05 value that has sometimes been used in clustering designs [46]. Therefore, the clustering would not affect the outcomes of the intervention.

Table 2 shows descriptive analyses for both groups in the outcome variables (pre- and post-intervention). Repeated measures-ANOVA tests were carried out to evaluate whether any change in the Experimental Group was significantly different to the change in the Control Group.

**Figure 2:**
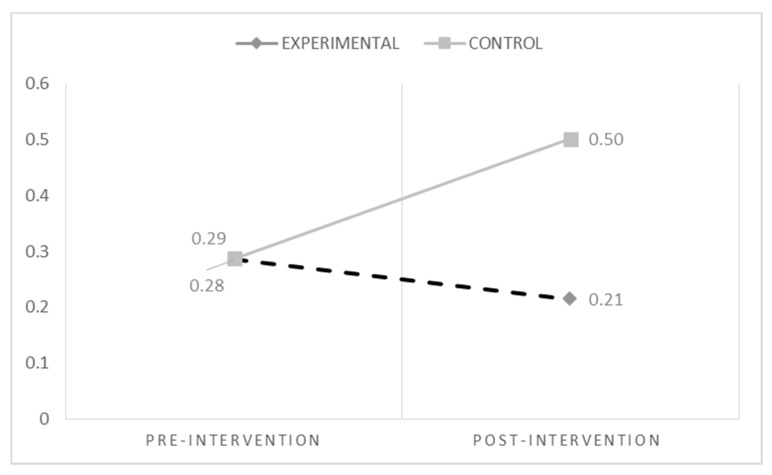
Changes in cyberbullying over time.

**Figure 3:**
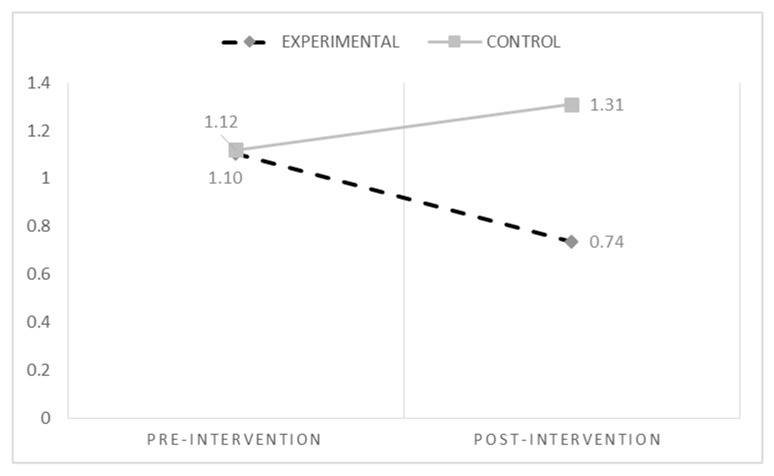
Changes in cybervictimization over time.

For cyberbullying (see Figure 2), the results showed a significant effect of the condition (Experimental vs. Control) (F_(1,620)_ = 4.10; *p* = 0.043) and a significant interaction time * condition (F_(1,620)_ = 6.46; *p* = 0.011). Bonferroni post hoc analyses indicated an increase of cyberbullying at T2 in the Control Group (F_(1,620)_ = 6.83; *p* = 0.009). Also, for cybervictimization (see Figure 3), the results showed a significant effect of condition (Experimental vs. Control) (F_(1,620)_ = 5.23; *p* = 0.022) and a significant interaction time * condition (F_(1,620)_ = 10.77; *p* = 0.001). Post hoc analyses indicated a decrease of cybervictimization at T2 in the Experimental Group compared with the Control Group (F_(1,620)_ = 13.71; *p* = 0.000).

**Figure 4:**
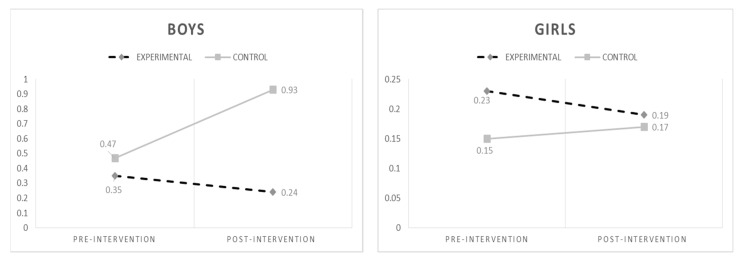
Changes in cyberbullying over time by gender.

**Figure 5:**
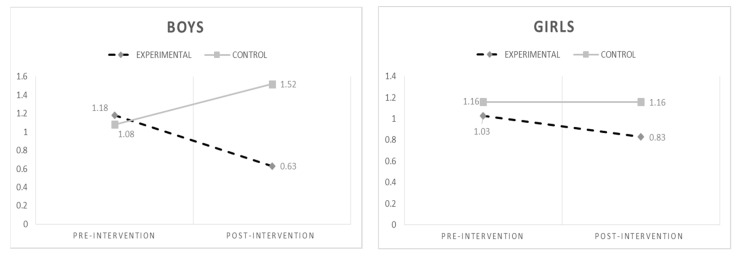
Changes in cybervictimization over time by gender.

We tested the effectiveness of the intervention for boys and girls. For this purpose, repeated measures ANOVAs were carried out separately for boys and girls. For cyberbullying (see Figure 4), the results showed a significant interaction time * condition (F_(1,1000)_ = 6.20; *p* = 0.013) among boys, but a non-significant interaction time * condition among girls (F_(1,1000)_ = 0.61; *p* = 0.44). Bonferroni post hoc analyses indicated an increase of cyberbullying at T2 among boys of the Control Group (F_(1,282)_ = 9.66; *p* = 0.002). For cybervictimization (see Figure 5), the results showed a significant interaction time * condition (F_(1,1000_) = 10.68; *p* = 0.001) among boys, but not for girls (F_(1,1000)_ = 1.28; *p* = 0.26). The results revealed that both cyberbullying and cybervictimization varied across groups and gender, indicating a significant decrease of both cyberbullying and cybervictimization over time among boys in the EG (but not girls). Post hoc analyses underlined a decrease in cybervictimization at T2 among boys for the Experimental Group (F_(1,282)_ = 11.14; *p* = 0.001).

## 4. Discussion

The present study aimed to present results of the effectiveness of the Tabby Improved Prevention Program (TIPIP), a multi-component program developed by combining the Ecological System Theory [30,31] and the Threat Assessment Approach [32,33,34,35]. We evaluated the short-term impact of the TIPIP on reducing cyberbullying and cybervictimization, by also looking at gender differences.

The results clearly show the efficacy of the TIPIP after six months of its extensive and thorough application, with reductions of cyberbullying and cybervictimization reported by students. The decreases in cyberbullying and cybervictimization in the Experimental Group has proved to be significant independently of student characteristics [20]. In particular, it is possible to assume that the inclusion of components of the program such as dedicated videos and cooperative work addressing cyberbullying and cybervictimization were those increasing its effectiveness [12].

With regard to the program’s impact on boys and girls, the results showed that the program is effective in reducing both cyberbullying and cybervictimization among boys. In particular, cyberbullying significantly decreased both among boys of the Experimental Group, while boys’ cyberbullying overall increased in the Control Group; for girls reported the same trend, the reduction was not significant. Similar to cyberbullying, the results showed a significant decrease in cybervictimization only among boys of the Experimental Group, while no significant differences were found among girls. However, it should be noticed that, even if not significant, a decrease in both cyberbullying and cybervictimization was observed among girls of the Experimental Group.

It is clear that the Tabby Improved Prevention and Intervention Program (TIPIP) works better for boys. These results are similar to those reported by Menesini and colleagues [28], who found a significant reduction of cyberbullying only among boys, and by Del Rey and colleagues [25], who found that the ConRed program was successful in reducing both cyberbullying and cybervictimization among boys. However, further studies are needed to understand whether this difference has to do with the components of the program, or on the role of other dimensions here not analyzed that might mediate such an effect such as empathy or moral disengagement [25,47].

We believe that one of the main strengths of this intervention program lies in the comprehensive cyberbullying and cybervictimization multicomponent theoretically driven approach. Furthermore, the activities undertaken with students were all planned to include curricula on classroom rules and cooperative group work, all elements that have been proven to be effective in preventing school bullying [10,11,12,13,29,48,49,50,51,52]. Also, all steps of the program were delivered, monitored and controlled one by one by the researchers, in cooperation with teachers and students. This reduces a risk of those programs where just a few days presence of an expert is provided as part of the program and material is distributed without any supervision or implementation check.

## 5. Conclusions

The current study has certain limitations. As in the majority of educational research, straightforward randomization is not always possible, and students were allocated to the conditions via their class [46]. Classes were randomly allocated to the research conditions by the researcher (to avoid possible teacher selection bias) and the Intraclass Correlation Coefficient was calculated. Possible contamination effects due to students of Experimental and Control Groups talk to each other were handled by the first author, which carried out all the activities with students of the Experimental Group. We cannot exclude, however, that some students from different groups talked to each other, although we did include this question at the end of the questionnaire and no contamination appeared from the analyses of the responses. However, we believe that due to the nature of the activities undertaken, that are most related to group work, negotiating and sharing common definitions and knowledge, the possible and simple word of mouth would not have affected the effectiveness of the program in its final results. A second possible limitation of the present study is related to the sole use of self-reported measures. In fact, despite their advantages [53], students could under-report their involvement in cyberbullying and/or cybervictimization or they could answer in a socially desirable manner [54]. As suggested by Topcu and Erdur-Backer [55], to overcome this limitation, multiple sources of information (for example peer, teacher and parent reports) could be used to investigate cyberbullying and cybervictimization. A third limitation concerns the short time of the follow-up measure (six months) and the lack of a long-term follow-up. According to the standards of evidence of prevention science [56], to claim that a program is effective, it would be necessary to report program efficacy in at least one long-term follow-up. Finally, repeated Anovas measures to test the program efficacy in reducing cyberbullying and cybervictimization for boys and girls as these are somehow skewed. However, even if our data were not normally distributed, we believe, consistent with Norman [57], that due to our sample size ‘*the means would be approximately normally distributed regardless of the original distribution*’.

Despite the aforementioned limitations, to the best of our knowledge, the present study is the first one aimed at investigating the effectiveness of a holistic, theoretically based cyberbullying and cybervictimization prevention program developed by combining the Ecological System Theoryand the Threat Assessment Approach, and the use of an actuarial self-reported instrument which has vast potential of use. We can conclude and show that this program and its components and its procedure is worth extending and promoting as a promising solution to address cyberbullying and cybervictimization as a vicious public health concern.

## Figures and Tables

**Table 1:** Descriptive statistics for the sample.

Age	Answer Criteria	Overall (622)	Boys (286)	Girls (336)	OR (C.I.)
		M = 12.14 (SD = 1.44)	M = 12.11 (SD = 1.44)	M = 12.18 (SD =1.44)	
Presence of social network profile(s)	At Least One	29.4%	31.7%	27.0%	0.71 * (0.54–0.94)
Personally know friends on social network	Only half	7.2%	6.8%	7.5%	1.01 (0.87–1.16)
Parents talk with students about Internet Safety	Never	18.6%	11.0%	4.8%	1.37 *** (1.16–1.62)
Parents control students’ online activities	Never	33.2%	29.7%	19.9%	1.48 *** (1.24–1.75)
Teachers talk with students about Internet Safety	Never	34.3%	18.7%	15.0%	1.01 (0.87–1.16)
Hours per day online	2–4 h	35.7%	41.9%	30.2%	0.83 * (0.70–0.97)
School achievement	Below average	7.6%	9.5%	6.0%	1.19 * (1.01–1.41)
School bully	At least sometimes	20.6%	28.2%	14.1%	2.45 *** (1.64–3.65)
School victim	At least sometimes	47.7%	53.2%	43.0%	1.49 * (1.08–2.04
Cyberbully	At least once	15.0%	21.5%	9.6%	2.59 *** (1.63–4.11)
Cybervictim	At least once	43.9%	43.0%	45.1%	0.92 (0.67–1.26)

Notes: * *p* < 0.05, *** *p* < 0.001, OR = Odds Ratio, C.I. = Confidence Interval.

**Table 2:** Pre–post differences in outcome variables.

Cyberbullying	Group	Pre	Post	Cyberbullying	Gender	Group	Pre	Post
		M (SD)	M (SD)				M (SD)	M (SD)
	EG	0.29 (0.79)	0.21 (0.61) ^a^		Boys	EG	0.35 (0.79)	0.24 (0.65) ^c^
	CG	0.28 (0.84)	0.50 (1.78)				0.47 (1.08)	0.93 (2.55)
					Girls	CG	0.23 (0.79)	0.19 (0.58) ^d^
							0.15 (0.57)	0.17 (0.58)
Cybervictimization	EG	1.10 (2.11)	0.74 (1.27) ^b^	Cybervictimization	Boys	EG	1.18 (2.17)	0.63 (1.12) ^e^
	CG	1.12 (1.73)	1.31 (2.34)			CG	1.08 (1.75)	1.52 (2.92)
					Girls	EG	1.03 (2.07)	0.83 (1.40) ^f^
						CG	1.16 (1.72)	1.16 (1.77)

Notes: EG = Experimental Group; CG = Control Group. ^a^ F _(1,620)_ = 6.46; *p* < 0.05; ^b^ F_(1,620)_ = 10.77; *p* < 0.001; ^c^ F_(1,1000)_ = 6.20; *p* < 0.05; ^d^ F_(1,1000)_ = 0.61; *p* > 0.05; ^e^ F_(1,1000)_ = 10.68; *p* < 0.001; ^f^ F_(1,1000)_ = 1.28; *p* > 0.05.
